# Support for learning in the perspective of patient safety in primary health care**^1^**

**DOI:** 10.1590/1518-8345.0784.2771

**Published:** 2016-08-18

**Authors:** Thatianny Tanferri de Brito Paranaguá, Ana Lúcia Queiroz Bezerra, Gabriela Camargo Tobias, Suely Itsuko Ciosak

**Affiliations:** 2 PhD, Adjunct Professor, Faculdade de Ciências da Saúde, Universidade de Brasília, Brasília, DF, Brazil.; 3 PhD, Professor Associado, Faculdade de Enfermagem, Universidade Federal de Goiás, Goiânia, GO, Brazil.; 4 Doctoral Student, Instituto de Patologia Tropical e Saúde Pública, Universidade Federal de Goiás, Goiânia, GO, Brazil.; 5 PhD, Associate Professor, Escola de Enfermagem, Universidade de São Paulo, São Paulo, SP, Brazil.

**Keywords:** Patient Safety, Knowledge Management, Primary Health Care, Quality Indicators in Health Care

## Abstract

**Objective:**

to analyze the support for learning, in the perspective of patient safety, offered in the work environment, according to health professionals working in primary care.

**Method:**

a transversal study, held with 86 health professionals working in primary care. A validated instrument was used, applied via the Internet. Descriptive statistical analysis was undertaken with a presentation of median, mean, standard deviation and coefficient of variation.

**Results:**

points which are favorable to supporting learning were evidenced, such as mutual respect, autonomy for organizing the work and valorization of new ideas, which obtained means above 7.0. The variables which hinder the process of learning in the work environment, perceived by the professionals, were: resistance to changes, and excess of work impeding reflection on how to improve the work, with means above 6.0.

**Conclusion:**

the study found evidence of indicators related to the process of staff development in the area of health and indicates the influence of support for learning for the improvement of the work processes and of patient safety. It is necessary that a culture involving the systematic assessment of educational interventions in health should be established, the aim being to diagnose actions which are more incisive for changing health professionals' attitude and, therefore, clinical practice.

## Introduction

Investigations into patient safety have been centered on hospital care, due to the greater technological density and to the innumerable risks in these environments. However, it is known that most care is provided by primary health care services (PHS)^1^, which can also result in accidents if not guided by minimum criteria of quality and safety. 

One Canadian study indicates that 31% of adverse events detected during inpatient treatment occur prior to admission, it being possible that they may have taken place in PHS^2^. The estimate for these events in the literature varies from 0.004 to 240 per 1000 consultations, and approximately 45% to 76% are avoidable^3^. According to the United Kingdom's Health Foundation, approximately 1% to 2% of consultations undertaken in PHS may cause incidents; and, considering that 90% of the health professional-patient relations are centered in the PHS, the number of incidents in this model of care may be superior to the estimates for the hospital context^4^.

Considering that the PHS is the gateway to the health system and that it has the premise of resolving approximately 85% of the population's health problems^5^, its professionals must work assertively. This reality reflects the importance of offering effective learning support, an issue which continues to be studied little in the health area, and which consists of an organizational culture which favors the undertaking of educational interventions, with the aim of provoking changes in individuals' behavior, to improve the interactions which occur in the work environment^6^. 

Educational interventions in health have, as their main objective, to promote knowledge and skills for the professionals such that these may attend the patients' real needs and thus improve the clinical quality and the outcomes of the care^7^. However, if there is no movement favorable to the construction of knowledge, regardless of the educational program undertaken, change in the scenario where the healthcare takes place may be inviabilized. 

Considering that the evaluation of the support for learning may guide the decision-making of the managers responsible for the health team's development and professional improvement, the present study aims to analyze the support for learning, in the perspective of patient safety, offered in the work environment, according to the health professionals who work in primary care. 

Its relevance lies in diagnosing indicators which are favorable and unfavorable for the dissemination of knowledge and making it possible to guide actions which promote changes in the work environment and in the health professionals' attitudes, for the sake of safe and quality care, contributing to the strengthening of this care model, which was created to put into effect the principles of the Unified Health System. 

## Method

A transversal study undertaken between June and July 2014, which used a self applied questionnaire, sent by email, to all the health professionals linked to the PHS network in Goiânia in the State of Goiás (GO), registered with *Telessaúde Goiás* ('Goiás Telehealth'). A total of 501 questionnaires were sent, and returns were received from 86 professionals, corresponding to an acceptability of 17.2%. 

The Internet questionnaire was made up of two parts. The first investigated the health professionals' profile, was made by the researchers, and was assessed for clarity and objectivity. The second was a validated assessment scale^8^_,_ which investigates the support for learning perceived in the work unit, encouraged by the immediate managers and by the work colleagues. On a scale of 0 to 10, the professionals judged 38 statements related to the support offered for learning, in which 0 corresponded to 'never', and 10, to 'always'. The higher the score attributed, the greater was the health professional's perception regarding the presence of the item in the work environment. The lower the score, the lower the perception of the item in the work environment. 

Descriptive statistical analysis was made with presentation of median, mean, standard deviation and coefficient of variation. The greater the interval of the coefficient of variation, the greater was the difference of opinion in relation to the presence of the item in the work environment. 

The ethical aspects complied with the provisions in the Resolution of the National Health Council N. 466/2012. The study was linked to the research project entitled "Impact of an interactive program in patient safety through e-learning, in the work process of basic health centers and the family health strategy, linked to Telehealth", approved by the Ethics Committee under Protocol N. 630.266/2014 with the support of of the National Council for Scientific and Technological Development (CNPq). 

## Results

A total of 86 professionals, from various professions linked to the health services of the PHS, participated in the study. Table 1 presents this population's general characteristics. 

**Table 1:** Characterization of the professionals of the primary health care network of the municipality of Goiânia, linked to Goiás Telehealth. Goiânia, GO, Brazil, 2014

Professional Characterization		N	%
Age			
	21 to 30 years old	34	39.5
	31 to 40 years old	25	29.1
	41 to 50 years old	16	18.6
	51 or over	11	12.8
Educational level			
	Senior high school complete	02	2.3
	Technical course	10	11.6
	Degree	19	22.1
	Specialist	40	46.5
	M.A	14	16.3
	Ph.D	01	1.2
Role			
	Care	50	58.1
	Management	25	29.1
	Care and management	11	12.8
Type of employment			
	State employee	58	67.4
	Contract	28	32.6
Monthly income*			
	Up to 3 minimum salaries	23	26.7
	3 to 6 minimum salaries	18	20.9
	6 to 9 minimum salaries	18	20.9
	9 to 12 minimum salaries	12	14.0
	Over 12 minimum salaries	15	17.4

*Minimum salary valued at R$ 724, referent to 2014, Brazil

The mean age was 36.5 years old (± 10 years), with the age range of 21 to 40 years old predominating for 68.6% (59) of the professionals. Higher education and the maximum title of specialist were more frequent, with a proportion of 86.1% (74) and 46.5% (40), respectively. The mean time since qualification was 11 years (± 9.5 years), that of professional experience was 8.3 years (± 7.7 years) and that of work in the current health unit was 4.6 years (± 5.8 years). The role of 'care' was predominant for 58.1% (50) of the professionals, and the type of employment mentioned by 67.4% (58), was 'state employee'^*^. Monthly income of up to three minimum salaries was confirmed by 26.7% (23) of the professionals, followed by 3 to 6 minimum salaries and 6 to 9 minimum salaries, both with 20.9% (18) of the responses. 

The professional category which most participated in this study was the nurses, with 44.2% (38), followed by dentists with 17.4% (15) and doctors, with 16.3% (14). The number of jobs worked varied, and 46.5% (40) of the professionals mentioned working in two or more jobs^**^. Qualification in public health was mentioned by 40.7% (35) of the professionals and training in patient safety was mentioned by 11.6% (10). The professionals' weakness in relation to a specific qualification regarding patient safety was evidenced, as only 14.0% (12) of the professionals stated that they had received training regarding patient safety in the health unit; and 88.4% (76) stated that they had not undertaken any training course in this area. 

Considering that building knowledge in the context of the practice requires organizational encouragement, the PHS professionals' perception regarding support for learning in the unit where they worked was investigated. This is shown in Tables 2, 3 and 4. 

**Table 2:** Support for learning offered in the unit where they worked for the primary health care professionals of the municipality of Goiânia, GO, Brazil, 2014

In the unit where I work	Median	Mean	Standard deviation	Coefficient of variation
Each member is encouraged to show what he or she thinks	6.0	6.5	2.8	0.43
There is autonomy to act without consulting the manager(s)	5.0	5.1	2.6	0.51
There is competition between the members	5.0	4.2	2.9	0.69
There is time set aside for seeking new ways of undertaking the work	5.0	4.7	3.4	0.72
The excess of work impedes participation in training	5.0	4.9	3.1	0.63
New ideas are valued	7.0	7.0	2.4	0.34
There is mutual respect	8.0	7.6	2.1	0.28
The excess of work impedes reflection on how to improve the work	6.0	5.7	2.8	0.49
There is autonomy questioning the orders given by the manager(s)	5.0	5.3	2.8	0.53
There is exchanging of information and knowledge regarding the application of new skills	7.0	6.6	2.5	0.38
There is tolerance for errors when one is trying to apply new skills	6.0	5.9	2.5	0.42
There is autonomy for organizing the work	8.0	7.2	2.2	0.31
There is openness to criticism when somebody applies new skills	7.0	6.5	2.3	0.35
There is resistance to change	6.0	6.2	2.4	0.39
Attempts to apply new skills are ignored	5.0	4.7	2.4	0.51
There is acceptance of the risks associated with applying new skills	6.0	5.8	2.1	0.36
There is encouragement to seek new learning	7.0	5.6	2.8	0.50
The tasks facilitate the application of new skills	6.0	6.1	2.5	0.41

**Table 3:** Support for learning offered by the immediate manager to the primary health care professionals of the municipality of Goiânia, GO, Brazil, 2014

My immediate manager(s):	Median	Mean	Standard deviation	Coefficient of variation
Establishes work objectives which encourage me to apply new skills.	7.0	6.2	2.5	0.40
Encourages me to seek new solutions to work problems	7.0	6.5	2.7	0.42
Encourages me to apply new skills	7.5	6.6	2.6	0.39
Values my suggestions for change	7.0	6.5	2.7	0.42
Accepts with me the risks of trying new ways of carrying out the work	7.0	6.3	2.9	0.46
Takes my ideas into account when they are different from his/hers	6.5	6.0	2.8	0.47
Limits the use of my new skills in the work	4.0	4.0	2.8	0.7
Encourages me to face challenges in the work	7.0	6.5	2.7	0.42
Praises me when I apply new skills	6.5	6.3	2.7	0.43
Removes difficulties and obstacles to the application of my new skills in the work	6.0	5.8	2.6	0.45
Ignores the changes which I propose as a result of what I have learned in training	4.0	4.1	2.8	0.68
Gives me the freedom to decide how to carry out my tasks	7.0	6.8	2.4	0.35
Is available to clear up my doubts regarding the use of new skills	8.0	6.8	2.8	0.41

**Table 4:** Support for learning offered by the work colleagues to the primary health care professionals of the municipality of Goiânia, GO, Brazil, 2014

My work colleagues:	Median	Mean	Standard deviation	Coefficient of variation
Give me advice when I have difficulties in applying new skills	8.0	7.8	2.2	0.28
Praise me when I apply my new skills	8.0	7.2	2.4	0.33
Encourage me to seek new knowledge directed towards the work	7.0	7.1	2.3	0.32
Support the attempts which I make to use new skills, which I learned in training, at work	7.0	7.2	2.1	0.29
Criticize me negatively when I make mistakes upon applying new skills at work	3.0	3.4	2.5	0.74
Encourage me to propose new ideas for the undertaking of the tasks	7.0	6.8	2.2	0.32
Feel threatened when I apply new skills at work	4.5	3.8	2.8	0.74

When the internal consistency of the scale used was analyzed, an alpha Cronbach of 0.8 was obtained, which attests good reliability in the responses obtained by the professionals of the PHS regarding support for learning offered in the unit where they work. 

The scores for the perception of the items related to support for learning present in the work environment varied from 4.2 to 7.6. The large interval of the coefficient of variation (0.31 to 0.72) indicated divergence of opinions in relation to the support offered. 

Among the variables which are favorable to the support of learning perceived most by the PHS professionals, emphasis is placed on mutual respect, autonomy for organizing the work and the valuing of new ideas, which obtained scores above 7.0, with a median between 7.0 and 8.0. However, the perception regarding the existence of time for seeking new knowledge, which obtained a mean score of 4.7, was relatively low. 

Among the variables which hinder support for learning, the most perceptible in the work environment was resistance to change, with a mean of 6.2 and a median of 6.0, as well as excess of work impeding reflection regarding how to improve the work, with a mean of 5.7 and a median of 6.0. However, on analyzing this excess of work as a factor impeding the professional from participating in training, the score was relatively low, obtaining a mean of 4.9. 

Even in the face of these contradictions, the tolerance for errors and acceptance of risks when trying to apply new skills are perceptible to the professional; their scores obtained means of 5.9 and 5.8, respectively. The mean of 6.5 was obtained in the item evaluating openness to criticism when somebody applies new skills, and indicated a movement towards the existence of support for learning in the work environment. 

In the analysis of the internal consistency of the scale for assessing support for learning offered by the immediate manager, an alpha Cronbach of 0.9 was obtained, which indicated excellent reliability. Variables which hinder the support for learning, such as limiting new skills and ignoring changes proposed based on knowledge acquired in training obtained scores of 4.0 and 4.1, and indicated that although little present, this conduct continues to be adopted by the immediate managers, in the perception of some PHS professionals. 

The encouragement to seek new solutions to the work problems is perceived by the professionals, and obtained a mean of 6.5 and a median of 7.0. This aspect agrees with the results of the analysis of the valorization of suggestions for change and encouragement to apply new skills which obtained the same mean and median scores. 

The health professionals also perceived the manager's support for applying new skills, as these accept the risks of new ways of carrying out the work and are available to clear up doubts. However, the coefficient of variation above 0.4 for both the variables indicates that these aspects must be worked upon more incisively among the professionals who do not have the same perception. 

Praise upon the application of new skills is delivered by the immediate manager, with a mean of 6.3 and median of 6.5. However, the mean of 5.8 and median of 6.0 were obtained upon assessing the removal of difficulties and obstacles by the immediate manager when faced with the application of new skills, which indicates a moderate perception of these variables. 

In the assessment of the support for learning offered by the PHS work colleagues, the analysis of internal consistency indicated an alpha Cronbach of 0.7, which corresponds to acceptable reliability. The mean of the items favorable to support for learning varied from 6.8 to 7.8, with a median between 7.0 and 8.0 and coefficient of variation between 0.28 and 0.33. The health professionals' strongest perceptions related to receiving advice from colleagues when faced with difficulties in applying new skills, and praise received, with medians of 8.0 and means above 7.0. 

It is highlighted that negative criticisms in the event of an error resulting from the application of new skills at work, and the feeling of being threatened by colleagues when new knowledge is applied were perceived little, obtaining scores below 4.0. 

## Discussion

The analysis undertaken made it possible to investigate the profile of the PHS health professionals and their perceptions regarding support for learning offered in the work environment. 

The professionals working in the PHS have a varied profile in relation to age range, to academic titles, and to the nature of their employment link with the health unit, among other characteristics which may favor the processes of knowledge management. This diversity is also observed in studies undertaken with professionals who work in the PHS in the other regions of Brazil^9^ ^-^ ^11^.

Considering the context of the PHS, in spite of the predominance of the 'state' employment link, it was ascertained that more than 30% of the health professionals are on temporary contracts. This type of system favors staff turnover, a characteristic which is not compatible with the principles of this model of care. In small municipalities, the proportion of contracts in the PHS can reach 96.7%^9^, which entails the need to assess the process of selection and recruitment of personnel undertaken. 

The time since qualification, length of professional experience and length of work in the health unit was variable, indicating that, simultaneously, one has more experienced professionals, who can contribute with their colleagues in the routine aspects of the unit, and the younger professionals, who can help in the use of new technologies, due to the fact that they left academia more recently, favoring the continuous transference of knowledge between the professionals. 

This scenario has required the implementation of models of training and of management of the workforce based on the competence of the professional, and requires workers with a differentiated profile, bearing in mind that the acquisition of knowledge occurs in distinct times and spaces^12^. Professional qualification is configured as an important attribute for better performance on the part of the health services and must be paired to a policy of valuing staff and reducing their turnover, with the objective of favoring the changing of attitudes and of the environment. 

The literature indicates that, in the organizational context, working upon the issue of patient safety has been complex and the construction of an environment which converges towards a "non-blame" culture with the recovery of the incident, and without fear of punishment, can create tensions among those involved^13^. It is ascertained that curricular training in patient safety on undergraduate courses in health occurs in a fragmented way, limited to specific skills, and clashes with traditional proposals, requiring a great need to elaborate methods and develop exclusive professional competences to reduce and prevent errors in health^13^ ^-^ ^14^. 

With weaknesses which originated since qualification, education at work, in the context of patient safety, becomes a challenge and must be encouraged so as to resolve gaps in the knowledge. The development of efficacious knowledge management in the health services is advised, so as to transfer to the professionals' context the best scientific evidence which can ensure the improvement of the clinical practice^15^.

Among the variables which are favorable to supporting learning in the PHS, mutual respect, autonomy for organizing the work and valuing of new ideas were the most perceptible by the health professionals, obtaining means above 7.0. The literature indicates a proportional correlation between support for learning and performance in the job, which is associated with undertaking activities efficiently, meeting the organizational expectations^16^. Emphasis is placed, therefore, on the positive influence between the variables of support offered to learning and the impact of the educational interventions in the work environment, which reinforces the power of the organizational context in the process of staff development.

The component of autonomy is considered one of the most important for promoting satisfaction among the professionals^17^. As a result, it is possible that the professional who perceives her autonomy in the work environment can become a proactive agent, capable of creating, reporting, discussing and, consequently, valuing quality care. 

In relation to the support for learning offered by the managers, a mean of 6.2 was observed for the encouragement of the health professionals in applying new skills, which must be encouraged by management which seeks the continuous improvement of the health care. However, a large proportion of the professionals asserted that the immediate manager never inhibits the use of new skills and, further, shoulders possible risks related to new ways of carrying out the work, which facilitates changing the context of the care. This support is important and must be linked to a systematized process for the control of these possible risks, so as to avoid the occurrence of failures could result in harm to the patient. 

It is highlighted that for the prevention of risks it is necessary to identify and analyze their origin and to systematize the preventive measures proactively - and not merely when the errors occur. Establishing a system for risk management is a route to seeking better control and monitoring of the work processes^18^.

The support for learning offered by work colleagues obtained the best scores, with the mean varying between 6.8 and 7.8, with a median of 7.0 to 8.0. The characteristics of this support which were most perceptible were the receiving of advice from work colleagues when faced with difficulties in applying new skills and the praise received when these new skills are applied. The strength of the teamwork in an integrated form was evident in the perception of the health professionals and must be cultivated, bearing in mind that, working together in an articulated way, health teams extend their capacity for care, their ability to resolve health problems, and share the responsibility for improving the population's quality of health and life^19^.

Emphasis was placed on the professionals' low perception in relation to negative criticism relating to errors resulting from the application of new skills and to the feeling of being threatened felt by colleagues when new knowledge is applied. These aspects indicate a tendency favorable to the application of new knowledge which influences the adoption of safer practices. This perception must be transferred and spread among the health professionals in an attempt to increase motivation to become involved in educational programs. 

The best care practices are directly related to the success and to the effectiveness in the training and empowerment of the health professionals^20^. It is argued that the development of educational interventions is more significant when the processes which facilitate the learning in the work environment are understood^7^. 

In dealing with learning for the prevention of incidents, at all levels of healthcare, it is necessary to take a broader view, beyond one's own professional work, and to pay attention to the multiple factors which place at risk the safety of the patient in the process of being cared for. This requires a feasible plan for the improvement of the quality of the care, as well as the extending of the culture of safety in the PHS, with a view to training not only the health professionals, but also the patients, to recognize and manage the risks, providing an opportunity for the shared ability to deal with change in the context of healthcare^1^. 

The present study indicates that the organizational structure influences the learning and is able to promote - or not - a safe environment with greater possibilities for changing the context where the practice of the care occurs. The maintainance of a positive organizational culture, the efficacious partnership of the work and an atmosphere of learning with mutual support among those involved are fundamental if the sharing of knowledge is to prosper and exercise a positive influence on the improvement of the patient and of the healthcare^7^. 

As a result, investing in the organizational support for learning is a significant strategy for achieving the quality of the clinical practice and, consequently, improving patient safety. Studies of this nature are rare in the area of health, especially in the PHS. In spite of having been undertaken in a specific context of healthcare, the results obtained list indicators which are related to the process of knowledge management, which can be used in primary, secondary and tertiary care, as a new benchmark in the process of the health professionals' development. 

As limitations of this study, one has the low number of respondents and the fact that it consists of a general evaluation of organizational support, as it is possible to assess the support for specific educational interventions and identify different perceptions, depending on the origin and complexity of the intervention. In spite of these limitations, the study offers support for the improvement of the concept of support for learning and makes it possible to ascertain the consonance between the policies of education and work and what actually happens in the professional practice in health. 

Favoring an environment of the sharing of knowledge entails attitudinal and organizational change. It is hoped that this study may awaken interest in deepening scientific knowledge on this issue, so as to produce evidence for clinical practice, in particular, for those responsible for managing the process of staff development. 

## Conclusion

Among the variables favorable to support for learning, emphasis is placed on mutual respect, autonomy, the valuing of new ideas, encouragement on the part of the managers for the use and application of new skills, the accepting of possible risks in the work, the receiving of advice when faced with difficulties for applying new skills, and praise received from colleagues when new skills are applied in the work. Among the variables which hinder the process of learning, those most perceived were: resistance to change, excess of work, negative criticism from colleagues in the event of errors and the feeling of being threatened as a result of the application of new skills. It was observed that it is not enough to promote learning opportunities but that it is necessary to provide encouragement and to offer support so that learning may take place - and to evaluate the results obtained, aiming to ascertain whether the time invested contributed to the improvement and transformation of the work processes and personnel. 

The practical implications of the results obtained suggest that the development of educational actions, in the ambit of patient safety, should be assessed more systematically in health institutions, remedying not only the problems related to support for learning, but, also, those related to the reaction of the health professional and to the impact of the intervention in the work environment, focusing on improvement and optimization of the resources and efforts employed with a view to the reduction of unwanted events. 

## References

[1] Marchon GS, Mendes WV (2014). Segurança do paciente na atenção primária à saúde revisão sistemática. Cad Saúde Pública.

[2] Baker GR, Norton PG, Flintoft V, Blais R, Brown A, Cox J (2004). The Canadian Adverse Events Study the incidence of adverse events among hospital patients in Canada. CMAJ.

[3] Makeham M, Dovey S, Runciman W, Larizgoita I (2008). Methods an measures used in primary care. Patient safety research. Dunedin,.

[4] Ministerio de Sanidad y Consumo (2008). Estudio APEAS. Estudio sobre la seguridad de los pacientes en atención primaria de salud.

[5] Roncoletta AFT (2010). O impacto da Medicina de Família na graduação médica aprendizado centrado na continuidade e atenção primária. Mundo Saúde.

[6] Lebbon AR, Lee SC, Johnson DA. (2015). Feedback facilitates transfer of training with US Hispanic workers in a healthcare laundry linen facility. Inj Prev..

[7] Clark E, Draper J, Rogers J (2015). Illuminating the process enhancing the impact of continuing professional education on practice. Nurse Educ Today.

[8] Abbad GS, Sallarenzo LH, Coelho-Júnior FA, Zerbini T, Vasconcelos KT, Abbad GS, Mourão L, Meneses PPM, Zerbini T, Borges-Andrade JE, Vilas-Boas RL (2012). Suporte à transferência de treinamento e suporte à aprendizagem. Medidas de Avaliação em Treinamento, Desenvolvimento e Educação. Ferramentas para gestão de pessoas.

[9] Gonçalves CR, Cruz MT, Oliveira MP, Morais AJD, Moreira KS, Rodrigues CAQ (2014). Human resources critical factor for primary health networks. Saúde Debate.

[10] Marsiglia RMG (2011). Perfil dos Trabalhadores da Atenção Básica em Saúde no Município de São Paulo região norte e central da cidade. Saúde Soc.

[11] Ohira RHF, Cordoni-Junior L, Nunes EFPA (2014). Análise das práticas gerenciais na Atenção Primária à Saúde nos municípios de pequeno porte do norte do Paraná, Brasil. Ciênc Saúde Coletiva.

[12] Camelo SHH, Angerami ELS (2013). Competência profissional a construção de conceitos, estratégias desenvolvidas pelos serviços de saúde e implicações para a enfermagem. Texto Contexto Enferm.

[13] Steven A, Magnusson C, Smith P, Pearson PH (2014). Patient safety in nursing education Contexts, tensions and feeling safe to learn. Nurse Educ Today.

[14] Bohomol E, Cunha ICKO (2015). Teaching patient safety in the medical undergraduate program at the Universidade Federal de São Paulo. Einstein.

[15] Grimshaw JM, Eccles MP, Lavis JN, Hill SJ, Squires JE (2012). Knowledge translation of research findings. Implementation Science.

[16] Balarin CS, Zerbini T, Martins LB (2014). A relação entre suporte à aprendizagem e impacto de treinamento no trabalho. REAd.

[17] Siqueira VTA, Kurcgant P (2012). Satisfação no trabalho indicador de qualidade no gerenciamento de recursos humanos em enfermagem. Rev Esc Enferm USP.

[18] Hinrichsen SL, Campos MA, Possas LCM, Sabino G, Vilella TAS (2011). Gestão da Qualidade e dos riscos na segurança do paciente estudo-piloto. RAHIS.

[19] Pereira RCA, Rivera FJU, Artmann E (2013). The multidisciplinary work in the family health strategy a study on ways of teams. Interface.

[20] Bastos LFL, Ciampone MHT, Mira VL (2013). Asessment of evaluation of transference support and training impact on the work of nurses Rev. Latino-Am. Enfermagem.

